# Management of Aberrant Frenum by Z-plasty Procedure: A Case Report

**DOI:** 10.7759/cureus.51853

**Published:** 2024-01-08

**Authors:** Pavan Bajaj, Unnati Shirbhate, Chitrika Subhadaresanee, Shivani Thakre

**Affiliations:** 1 Department of Periodontics, Sharad Pawar Dental College, Datta Meghe Institute of Higher Education and Research, Wardha, IND

**Keywords:** ortho-perio, midline diastema, periodontal, frenum, mucogingival problems, plastic surgery, frenectomy, z-plasty

## Abstract

During or after the orthodontic closure, persistent diastemas are frequently the result of a high frenum attachment. A labial frenectomy is a complete removal of the frenum attachment, which typically attaches to the space between the upper two anterior teeth and the centre of the upper lip. It might be required if there is space between the teeth due to a frenulum positioned too high on the gums. Many surgical technique modifications, including Miller's technique, Z-plasty, and V-Y-plasty, have been established since the conventional classical frenectomy procedure was initially presented to cope with the difficulties associated with an aberrant labial frenum. This case report demonstrates that a Z-plasty approach was used to remove the 21-year-old female patient's high papillary-type labial frenum attachment and how orthodontic treatment led to the closure of the midline diastema. For several reasons, the frenectomy procedure with Z-plasty proved to be reliable and yielded outstanding aesthetic outcomes for the removal of the aberrant labial frenum connection. Understanding Z-plasty will enable primary intention-based tissue healing, reduce the risk of tissue contractures, shorten the patient's recovery, and enhance the patient's aesthetic outcomes.

## Introduction

Getting dental treatment is becoming increasingly important to have a beautiful smile due to aesthetic considerations. The muscle and connective tissue fibres that connect the cheek and lip to the gingiva, periosteum, and alveolar mucosa are found in the frenum, a mucous membrane fold. Attention to the frenum has become essential because one of the aetiological causes for the persistence of a midline diastema is the existence of an abnormal frenum [1]. Placek et al. established a clinical, morphological classification of maxillary frenum attachment based on the anatomic attachment site. They categorised frenum attachment into four types, depending on where the frenum is attached: in the interdental papilla-papillary, the connected gingiva-gingival, the mucogingival junction-mucosal, or via the interdental papilla up to the palate-papilla penetrating [2]. When the frena attach too firmly to the gingival border, either because of muscle strain or difficulty with plaque elimination, the health of the gingiva may be compromised. Mucogingival problems associated with gingival health can be primarily prevented by understanding the relationship between dental hygiene and the labial frenum attachment [3].

Therefore, frenectomy and frenotomy, two surgical treatments, can be used to manage such an issue. While a frenotomy involves making an incision and moving the frenal attachment, a frenectomy consists of removing the frenum entirely, including its attachment to the underlying bone. This process has been carried out using a variety of approaches up till now. In this instance, Z-plasty was used to accomplish the frenectomy. A plastic surgery procedure called Z-plasty enhances the aesthetic and cosmetic appearance of scarring. A central incision is made, and two equal-sized triangle flaps are created, transposed, and sutured [4].

## Case presentation

**Figure 1:**
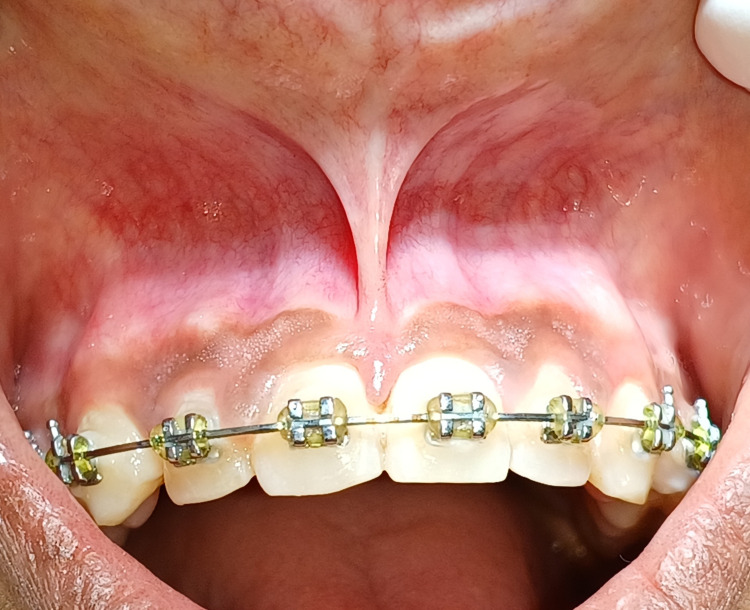
Pre-operative view of high labial papillary type frenal attachment.

The Department of Orthodontics referred a 21-year-old female patient to the Department of Periodontics and reported ongoing orthodontic treatment with pre-orthodontic midline diastema and its closure achieved during orthodontic treatment with high papillary type frenal attachment in between two maxillary central incisors as can be appreciated in Figure 1.

**Figure 2:**
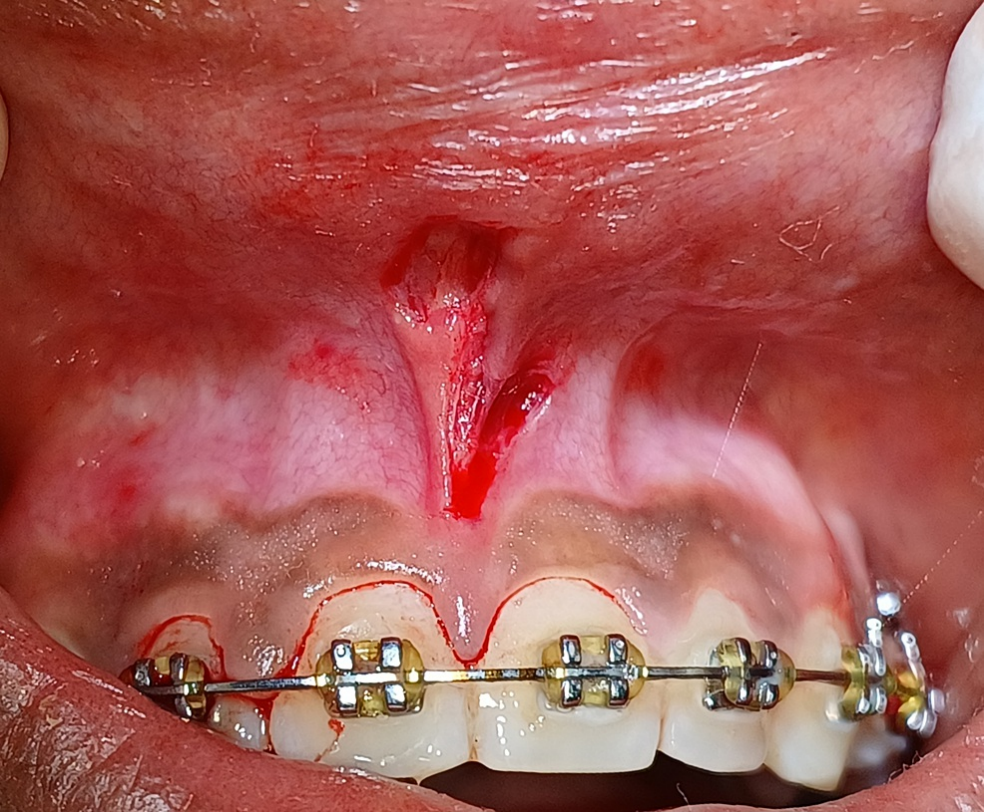
Z-shaped incision was made by two lateral horizontal incisions and one vertical incision.

In case of high frenal attachment associated midline diastema, labial frenectomy may be done before or during the procedure or even after following the orthodontic treatment. In this case, orthodontic midline diastema closure was planned in the initial instance, and after that, a frenectomy procedure was performed. A comprehensive medical history was obtained to exclude non-significant conditions. The patient was advised about the necessary surgical operation and received informed written consent. A routine haematological examination was performed, and the results were within the normal range. Following oral prophylaxis, surgical removal of the frenum was carried out under local anaesthesia, with all aseptic conditions and precautions followed. The Z-shaped incision in Figure 2 was created by making two lateral horizontal incisions at the apex and base at a 60° angle and one central mid-vertical incision across the frenal attachment. This resulted in two triangular flaps of similar size and form.

**Figure 3:**
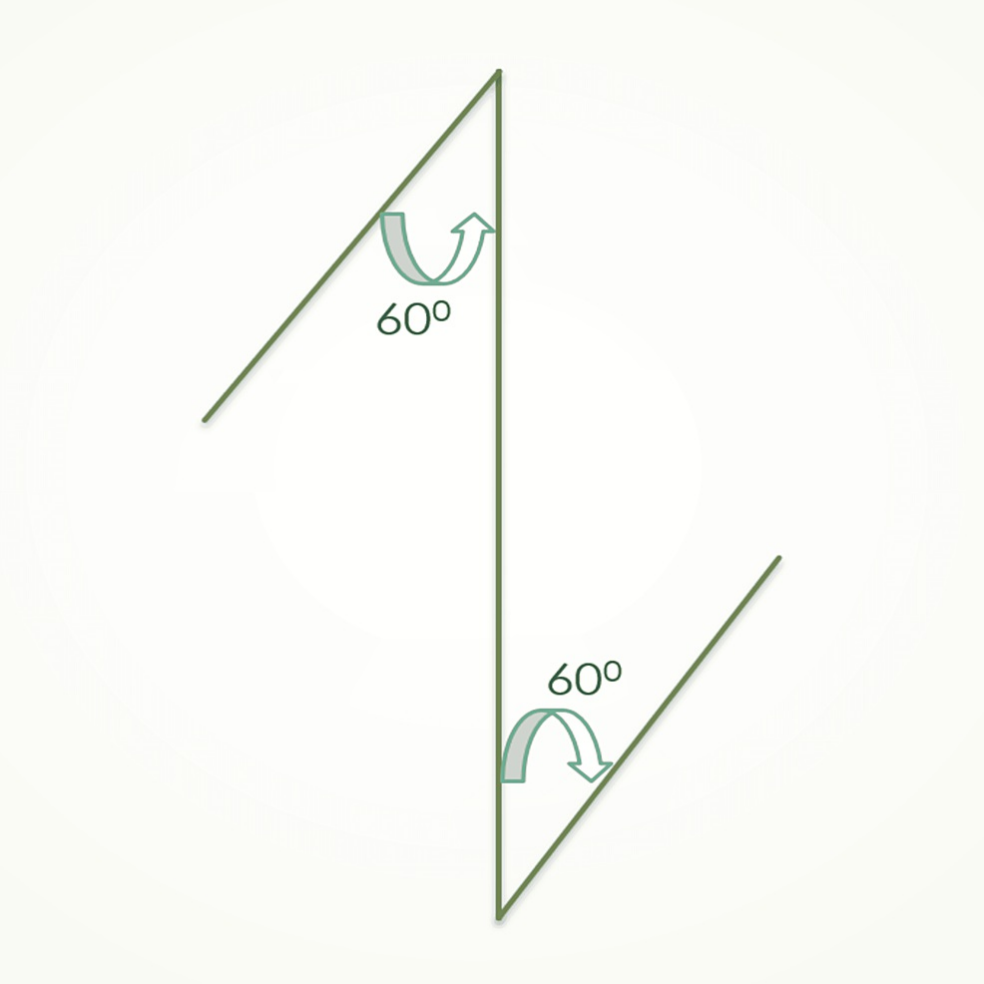
Diagrammatic representation of the Z-plasty frenectomy design. Figure Credit: Author Unnati Shirbhate

Each side of the Z-plasty frenectomy procedure design has a 60-degree angle. This angle determines the degree of tissue extension, which is illustrated in the diagrammatic representation in Figure 3.

**Figure 4:**
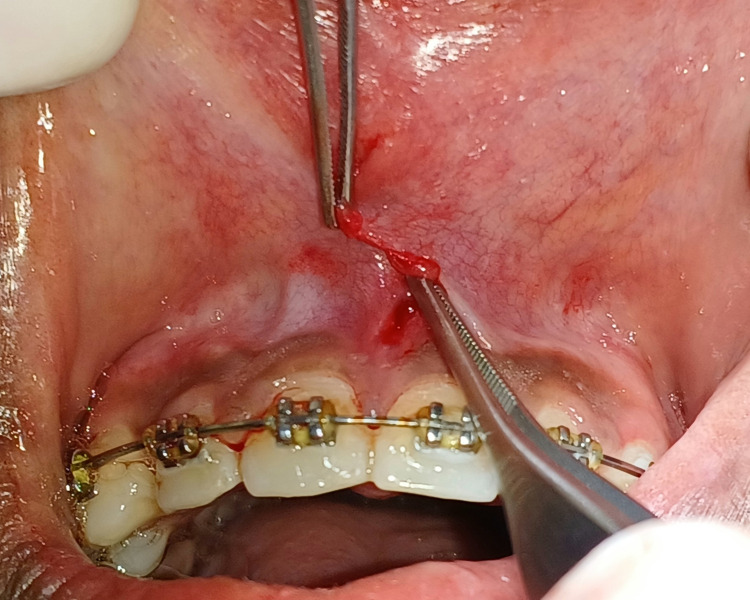
Two flaps were relocated to the side opposite each flap's apex.

A sufficient undermining of the peripheral tissues was performed to reduce the distortion of the underlying structures and facilitate adequate flap placement. Two flaps were relocated to the side opposite each flap's apex after achieving hemostasis, as seen in Figure 4.

**Figure 5:**
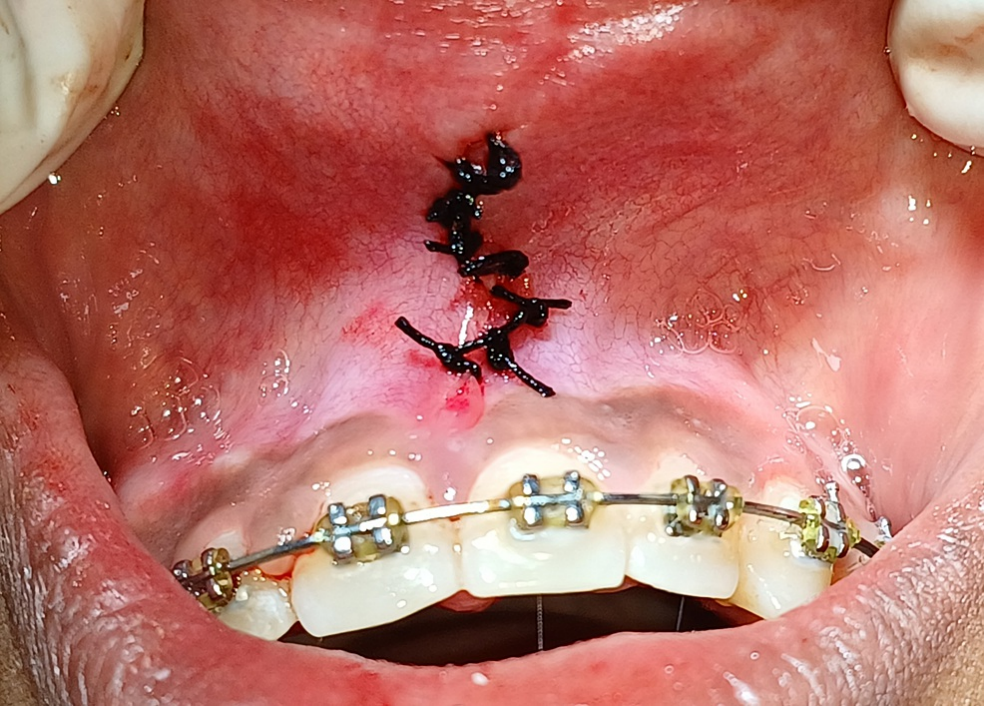
Sutures were placed by securing two opposing flaps.

After that, they were secured in the position shown in Figure 5 by suturing them to the defect on the other side of the flap base.

**Figure 6:**
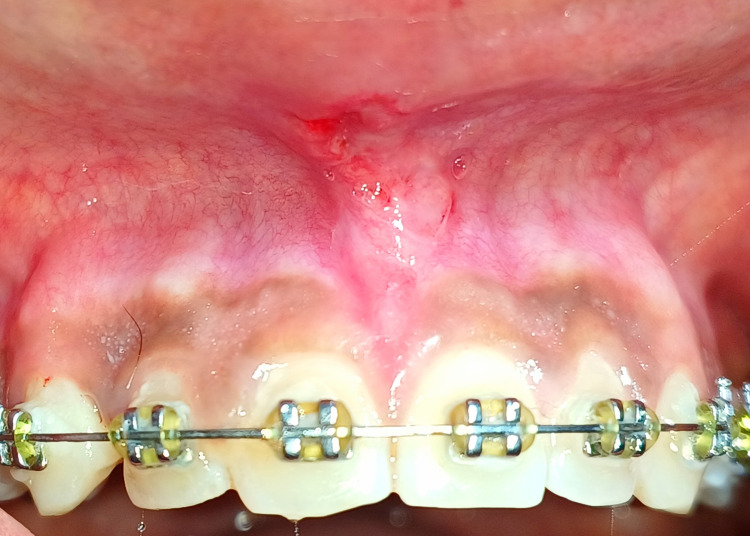
Postoperative view after suture removal showing satisfactory tissue healing of the surgical site.

The patient was reviewed for suture removal after seven days, revealing routine satisfactory healing and no discomfort as can be appreciated in Figure 6.

**Figure 7:**
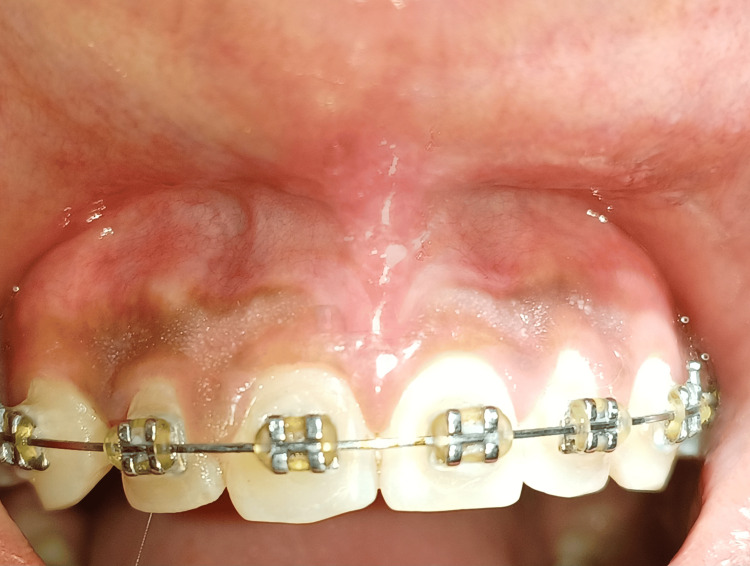
Completely satisfactory healing seen at one-month follow-up.

Labial frenectomy performed with Z-plasty procedure revealed complete satisfactory healing, no scar formation, and desirable results maintained in regard to ortho-perio consideration at one-month follow-up (Figure 7).

## Discussion

Classic frenectomy, Z-plasty, V-Y plasty, electrosurgery, and carbon dioxide laser are various methods used for frenectomy [5]. The Z-plasty concept and procedure have an unclear specific history; however, they most likely date back several centuries. Z-plasty was initially developed in 1856 by Denonvilliers to treat eyelid scars; today, it is applied to every area [6]. Even though each explanation varies and is widely used, some similar elements are essential to comprehending Z-plasty. Z-plasties can be done for three different reasons: 1) to thin out a scar, 2) to extend a scar or relieve a contracture, or 3) to realign a scar inside a relaxed skin tension line (RSTL) [5,6].

Z-plasty may yield an excellent cosmetic outcome [7]. This method can be used to reposition a scar to better align with the lines of most minor tension on the skin or a natural skin fold. Simple Z-plasty flaps are made with a 60-degree angle on either side. Scars from traditional 60° Z-plasty are 75% longer, but scars from 45° and 30° designs are 50% and 25% longer, respectively [8]. The 60-degree angles on either side provide the framework of the Z-plasty design, the more significant the angle, the greater the extension [7,9]. Because it aids in helping the skin repair along its natural lines by facilitating the re-distribution of tension on the skin and wound, the Z pattern is beneficial. It has a camouflaging effect and reduces the production of scars. As a result, the wound has less disturbance and improved healing due to the tissue tension being dispersed in multiple directions. Because it lengthens and diverts the tissue wound, it will lessen the formation of scars and aid in deepening the vestibule, which is impossible with other methods. Z-plasty frenectomy can result in flap necrosis, bleeding, infection, and sloughing of the flap from excessive wound tension. Nonetheless, a precise and cautious technique can avoid these difficulties [9]. To realign the frenulum and enhance results, Z-Plasty is a variation of the conventional frenectomy process [10].

## Conclusions

Z-Plasty frenectomy procedure comes under periodontal plastic surgery, which was performed for the removal of a high labial frenum attachment in the given case and demonstrated satisfactory uneventful postoperative healing and no hypertrophic scar development, which was satisfying for the patient's aesthetic and functional needs as well as the surgeons'. The papilla penetrating frenum demonstrated a satisfactory outcome and desirable results, with Z-Plasty providing functional and aesthetic clearance in the orthodontic treatment procedure despite the complexity of this technique. This is a sensitive cosmetic procedure; in the given case, it lead to desirable outcomes.
